# RNA-Seq Reveals Enhanced Sugar Metabolism in *Streptococcus mutans* Co-cultured with *Candida albicans* within Mixed-Species Biofilms

**DOI:** 10.3389/fmicb.2017.01036

**Published:** 2017-06-08

**Authors:** Jinzhi He, Dongyeop Kim, Xuedong Zhou, Sang-Joon Ahn, Robert A. Burne, Vincent P. Richards, Hyun Koo

**Affiliations:** ^1^State Key Laboratory of Oral Diseases, Department of Endodontics, West China Hospital of Stomatology, Sichuan UniversityChengdu, China; ^2^Biofilm Research Labs, Levy Center for Oral Health, Department of Orthodontics, School of Dental Medicine, University of Pennsylvania, PhiladelphiaPA, United States; ^3^Department of Oral Biology, College of Dentistry, University of Florida, GainesvilleFL, United States; ^4^Department of Biological Sciences, Clemson University, ClemsonSC, United States

**Keywords:** early childhood caries, biofilms, *Streptococcus mutans*, *Candida albicans*, transcriptome, RNA-Seq

## Abstract

Early childhood caries (ECC), which can lead to rampant tooth-decay that is painful and costly to treat, is one of the most prevalent infectious diseases affecting children worldwide. Previous studies support that interactions between *Streptococcus mutans* and *Candida albicans* are associated with the pathogenesis of ECC. The presence of *Candida* enhances *S. mutans* growth, fitness and accumulation within biofilms *in vitro*, although the molecular basis for these behaviors is undefined. Using an established co-cultivation biofilm model and RNA-Seq, we investigated how *C. albicans* influences the transcriptome of *S. mutans*. The presence of *C. albicans* dramatically altered gene expression in *S. mutans* in the dual-species biofilm, resulting in 393 genes differentially expressed, compared to mono-species biofilms of *S. mutans*. By Gene Ontology analysis, the majority of up-regulated genes were related to carbohydrate transport and metabolic/catabolic processes. KEGG pathway impact analysis showed elevated pyruvate and galactose metabolism, suggesting that co-cultivation with *C. albicans* influences carbohydrate utilization by *S. mutans*. Analysis of metabolites confirmed the increases in carbohydrate metabolism, with elevated amounts of formate in the culture medium of co-cultured biofilms. Moreover, co-cultivation with *C. albicans* altered transcription of *S. mutans* signal transduction (*comC* and *ciaRH*) genes associated with fitness and virulence. Interestingly, the expression of genes for mutacins (bacteriocins) and CRISPR were down-regulated. Collectively, the data provide a comprehensive insight into *S. mutans* transcriptomic changes induced by *C. albicans*, and offer novel insights into how bacterial–fungal interactions may enhance the severity of dental caries.

## Introduction

Biofilms are associated with many infectious diseases in humans, including those occurring in the mouth (Hall-Stoodley et al., 2004). Early childhood caries (ECC) is a highly prevalent and difficult to treat biofilm-dependent disease, aﬄicting mostly underprivileged children worldwide and resulting in estimated annual expenditures of more than $120 billion in the United States alone (Kassebaum et al., 2015). Children with ECC are heavily infected with *Streptococcus mutans*, due in large part to protracted feeding of dietary sugars, such as sucrose (Berkowitz et al., 1984; Palmer et al., 2010; Parisotto et al., 2010), which leads to rapid accumulation of virulent biofilms characterized by an exopolysaccharides (EPS)-rich and highly acidic milieu that cause rampant destruction of the teeth (Takahashi and Nyvad, 2011; Hajishengallis et al., 2017).

*Streptococcus mutans* has long been regarded one of the key etiologic agents of ECC. *S. mutan*s possesses an exceptional ability to produce EPS using dietary sucrose via secreted exoenzymes termed glucosyltransferases (Gtfs), as well as being robustly acidogenic and acid-tolerant (Marsh et al., 2011; Koo et al., 2013). In addition, *S. mutans* can efficiently cope with environmental stresses, which contributes to its ability to establish biofilms, to persist in the host, and to compete with other oral bacteria, particularly when conditions are conducive to initiation and progression of dental caries (Lemos and Burne, 2008). However, *S. mutans* does not act alone in cariogenic biofilms, as additional organisms can also contribute to the initiation and/or progression of caries (Tanner et al., 2011; Gross et al., 2012). Interestingly, results from several clinical studies reveal that *Candida albicans* is often detected in high numbers with *S. mutans* in biofilms from children with ECC (de Carvalho et al., 2006; Raja et al., 2010; Yang et al., 2012; Klinke et al., 2014; Qiu et al., 2015).

*Candida albicans* is a commonly detected opportunistic fungus in the oral cavity (Ghannoum et al., 2010). This organism interacts actively with commensal (viridans) streptococci and forms biofilms on acrylic and mucosal surfaces (Jenkinson and Douglas, 2002; Diaz et al., 2012) to cause oral mucosal infections (Thein et al., 2009; Xu et al., 2014). In contrast, *C. albicans* does not bind well to *S. mutans*, nor does it colonize teeth effectively on its own (Jenkinson et al., 1990; Gregoire et al., 2011). However, physical co-adhesion between *S. mutans* and *C. albicans* is markedly increased in the presence of sucrose (Branting et al., 1989; Gregoire et al., 2011; Metwalli et al., 2013; Falsetta et al., 2014). *S. mutans* Gtfs are capable of adhering to the surface of *C. albicans* and producing large amounts of EPS *in situ* using sucrose as substrate (Gregoire et al., 2011; Hwang et al., 2015). In turn, the EPS on the fungal surface promotes adhesive interactions and cross-kingdom biofilm development with *S. mutans* (Falsetta et al., 2014).

In biofilms formed *in vitro*, the presence of *C. albicans* dramatically modifies the physical environment by increasing biomass and EPS production, enhancing biofilm accumulation and stability (Falsetta et al., 2014). Furthermore, *C. albicans* appears to activate *S. mutans* genes associated with biofilm formation and genetic competence (Falsetta et al., 2014; Sztajer et al., 2014). Importantly, using a rodent model of the disease and a diet rich in sucrose, enhanced levels of *S. mutans* in plaque-biofilms were associated with co-infection with *C. albicans*, which lead to onset of rampant caries similar to ECC (Falsetta et al., 2014). However, the molecular pathways by which such interactions stimulate *S. mutans* growth/metabolism, accumulation and virulence remain unclear.

Recently, RNA sequencing (RNA-Seq) combined with integrated gene network-pathway analysis greatly enhanced annotation/detection of bacterial transcripts and interpretation of genomic data (Croucher and Thomson, 2010; Zeng et al., 2013), including in mixed-species biofilms (Dutton et al., 2016). Here, we investigate the impact of the presence of *C. albicans* on the whole transcriptome of *S. mutans* using RNA-Seq and systems analysis. We first optimized enrichment of *S. mutans* mRNA from bacterial–fungal mixed total RNA, and then used RNA-Seq to transcription profile enriched mRNAs from single- and dual-species biofilm. The results show that the presence of *C. albicans* dramatically altered the transcriptome of *S. mutans*. Gene Ontology (GO) and Kyoto Encyclopedia of Genes and Genomes (KEGG) pathway impact analyses supported that co-culturing of *S. mutans* with *C. albicans* enhanced carbohydrate metabolism by *S. mutans*. Carbohydrate and metabolites analysis confirmed increased sugar utilization and elevated levels of formate in the supernatant fluid of dual-species biofilms. Moreover, we also found that *C. albicans* alters the transcription of two-component signal transduction systems that are important for fitness and sucrose-dependent biofilm formation. Conversely, mutacin (bacteriocin) production was down-regulated, which could influence the bacterial composition of the biofilms formed when *C. albicans* is present. Collectively, this study provides new insights into the effects of an opportunistic fungus (*C. albicans*) on the expression of genes that are integral to the persistence and virulence of *S. mutans* and how these inter-kingdom interactions may modulate the pathogenic potential of biofilms in ECC.

## Materials and Methods

### Bacterial Strains and Growth Conditions

*Streptococcus mutans* strain UA159 serotype *c* [a cariogenic bacterial pathogen (genome sequence accession number AE014133)] and *C. albicans* SC5314 (genome sequence accession number CP017630) were used in the present study to generate single and dual-species biofilm. Both strains were stored at -80°C in tryptic soy broth containing 20% glycerol.

### Biofilm Preparation

Biofilms were formed using a saliva-coated hydroxyapatite (sHA) disk model, as described elsewhere (Koo et al., 2010; Falsetta et al., 2014). Briefly, the hydroxyapatite disks (1.25 cm in diameter, surface area of 2.7 ± 0.2 cm^2^; Clarkson Chromatography Products, Inc., South Williamsport, PA, United States) were coated with filter-sterilized, clarified whole saliva and vertically suspended in 24-well plates using a custom-made wire disk holder (Koo et al., 2010). For single-species biofilms, each disk was inoculated with approximately 2 × 10^6^ CFU/mL of *S. mutans* in ultrafiltered (10-kDa cutoff; Millipore, Billerica, MA, United States) tryptone-yeast extract broth (UFTYE; 2.5% tryptone and 1.5% yeast extract, pH 7.0) containing 1% sucrose (37°C, 5% CO_2_). For dual-species biofilms, approximately 2 × 10^4^ CFU/mL of *C. albicans* containing predominantly yeast cell forms was also added to the inoculum; the composition of the microorganisms in the inoculum is similar to that found in saliva samples from children with ECC (de Carvalho et al., 2006; Falsetta et al., 2014). During the first 18 h, the organisms were grown undisturbed so as to allow initial biofilm formation; the culture medium was then changed twice daily at 8 a.m. and 6 p.m. until the end of the experimental period (42 h).

### RNA Extraction and Purification

Biofilms were harvested after 42 h incubation. RNA was extracted and purified using protocols optimized for biofilms formed *in vitro* (Cury and Koo, 2007). Three separate biological replicates for each group (single and mixed-species) were performed. Briefly, disk sets were incubated in RNALater (Applied Biosystems/Ambion, Austin, TX, United States), then the biomass was removed from the sHA disks. RNAs were purified and treated with DNase on a column using the Qiagen RNeasy Mini kit (Qiagen, Valencia, CA, United States). The RNAs were then subjected to a second DNase I treatment with Turbo DNase (Applied Biosystems/Ambion) and were purified using the Qiagen RNeasy MinElute cleanup kit (Qiagen). RNAs were quantified using the NanoDrop ND-1000 spectrophotometer (Thermo Scientific/NanoDrop, Wilmington, DE, United States). RNA quality was evaluated using an Agilent 2100 bioanalyzer (Agilent Technologies Inc., Santa Clara, CA, United States), and all RNAs used for downstream experiments were determined to have RNA integrity numbers (RIN) of 9.5 and above.

### Bacterial mRNA Enrichment and RNA-Seq Performance

To deplete fungal total RNA, the MICROBEnrich^TM^ Kit (Ambion of Life Technologies, Grand Island, NY, United States) was used with modifications. Briefly, RNA was combined with binding buffer and capture oligonucleotide mix. The RNA mix was heated to 70°C for 10 min then incubated at 37°C for 1 h to hybridize to the capture oligos. The RNA/capture oligo mix was equilibrated with Oligo MagBeads and incubated at 37°C for 15 min. Tubes were placed on a magnet to separate the supernatant fluids containing the enriched bacterial total RNA from the Oligo MagBeads. The enriched bacterial RNA was purified and concentrated by ethanol precipitation. Ribo-Zero^TM^ rRNA Removal Kits for Gram-Positive Bacteria (Epicentre, Madison, WI, United States) and MICROBExpress^TM^ Kit (Ambion) were tested for their efficiency of depleting the bacterial ribosomal RNA according to the supplier’s specifications. (1) RNA input amounts determined the amount of Ribo-Zero rRNA removal solution to add (10 μL rRNA removal solution for 2.5 to 5 μg, or 8 μL for <2.5 μg total RNA per reaction). Samples in Ribo-Zero rRNA removal solution were incubated at 68°C for 10 min followed by a 15 min incubation at room temperature. To remove the hybridized rRNA molecules from the mRNA, the RNA/rRNA solution reactions were incubated with the prepared microsphere beads, mixed well and placed at room temperature for 10 min, then at 50°C for 10 min. The mRNAs were separated from the microspheres bound with rRNAs by a filter column provided in the kit. The final purification of eluted mRNA was performed by ethanol precipitation. (2) For MICROBExpress^TM^ Kit, RNA was mixed with binding buffer and capture oligonucleotide mix. The RNA mix was heated to 70°C for 10 min then incubated at 37°C for 15 min to hybridize the capture oligos. The RNA/capture oligo mix was equilibrated with Oligo MagBeads and incubated at 37°C for 15 min. Tubes were placed on a magnet to separate the supernates containing the enriched bacterial total RNA from the Oligo MagBeads. The enriched bacterial RNA was purified and concentrated by ethanol precipitation. The final quality of enriched bacterial mRNA samples was analyzed using an Agilent Bioanalyzer (Agilent Technologies, Santa Clara, CA, United States). The efficiency of Ribo-Zero^TM^ rRNA Removal and MICROBExpress^TM^ Kits were compared and the results are included in the Supplementary Figure S1. Based on experimental data, we selected MICROBEnrich + Ribo-Zero as an optimized method to enrich *S. mutans* mRNA from mixed bacterial–fungal RNA samples.

cDNA libraries were generated from the enriched mRNA samples using NEBNext Ultra directional RNA library prep kit for Illumina and NEBNext multiplex oligonucleotides for Illumina (New England BioLabs, Ipswich, MA, United States), following instructions from the supplier. RNA-Seq was performed on the NextSeq500 (75-bp single end reads) by the NextGen DNA Sequencing Core Laboratory of the Interdisciplinary Center for Biotechnology Research at the University of Florida (Gainesville, FL, United States). Read mapping was performed on a Galaxy server hosted by the high-performance research computing center at the University of Florida (HiPerGator2.0) using Map with Bowtie for Illumina (version 1.1.2). Reads were mapped to the *S. mutans* UA159 genome. Mapped reads were then counted using the Python script htseq-count (Anders et al., 2015).

### Statistical Analysis of RNA-Seq Data

Fold changes and significant differences in gene expression between growth conditions were calculated using the convergence of three separate approaches: DEseq, edgeR, and limma (Anders and Huber, 2010; Robinson et al., 2010; Law et al., 2014), as implemented in the R/Bioconductor package metaseqR (Moulos and Hatzis, 2015). GO terms were assigned to genes using Blast2GO v.2.5.0 (Gotz et al., 2008). Relative enrichment (overrepresentation) of GO terms for up-regulated genes compared to a background of GO terms for all genes was assessed using Fisher exact tests. The test was performed using the Gossip statistical package (Blüthgen et al., 2005) implemented within Blast2GO. The false discovery rate (FDR) procedure of Benjamini and Hochberg (1995) was used to correct for multiple hypothesis testing (FDR = 0.05).

To gain further insights into the effects of co-cultivation of *C. albicans* on *S. mutans*, we performed a KEGG pathway impact analysis using the software package Pathway-Express as implemented in the R/Bioconductor package ROntoTools (Calin and Draghici, 2016). A systems biology approach such as this has the advantage of being able to factor the complex interactions among genes. It combines evidence from traditional expression level data with information regarding the dynamics of gene–gene interaction and the relative position of the gene within a pathway. Positional information is important as the action of genes up-stream in a pathway can propagate further down-stream and amplify effects of changes in gene expression. By combining all evidence, pathway impact can be calculated. FDR procedure of Benjamini and Hochberg (1995) was again used to correct for multiple hypothesis testing (FDR = 0.05).

### Data Validation

To validate the RNA-Seq data, quantitative real-time PCR (qRT-PCR) was employed to measure changes in the amount of mRNA of selected genes. The cDNA was synthesized from 1 μg of purified RNA with Bio-Rad iScript cDNA synthesis kit (Bio-Rad Laboratories, Inc., Hercules, CA, United States), and quantitative amplification condition using Bio-Rad iTaq Universal SYBR Green Supermix and Bio-Rad CFX96 system (Bio-Rad Laboratories, Inc.). Standard curves for each primer were used to determine the relative number of cDNA molecules, and relative expression was calculated by normalizing to the *gyrA* gene transcripts, which is a validated reference gene for normalization of qRT-PCR (Rocha et al., 2015; Zeng and Burne, 2016). The minimum information for publication of qRT-PCR experiments (MIQE) guidelines were followed for quality control of the data generated and for data analysis (Bustin et al., 2009).

### Carbohydrates and Metabolites Analyses

The biofilm and the respective surrounding culture medium were collected at 42 h, homogenized via sonication and centrifuged at 5,500 × *g* for 10 min at 4°C. The supernatant was filtered through 0.2 μm-pore-size membrane filter (ultra-low protein binding, surfactant-free cellulose acetate, Nalgene, Rochester, NY, United States). The amount of sucrose, glucose, and fructose in the supernatant were quantified using high-performance anion-exchange chromatography (HPAEC; Dionex, Sunnyvale, CA, United States) and the biofilm-derived metabolites were identified and quantified through ^1^H nuclear magnetic resonance (^1^H-NMR; Bruker Avance III HD NMR spectrometer, Bruker Biospin, Billerica, MA, United States) as described previously (Kim et al., 2017). The significance was determined by direct comparison with concentration in the blank (original UFTYE medium) and substrates and metabolites are characterized by the calculation of fold changes (log_2_) relative to the blank (UFTYE medium). The concentrations of glucose, fructose, formate, and lactate in the supernatant of single- and dual-species biofilms were subtracted with the values of blank. A pairwise comparison (non-parametric Mann–Whitney *U* test or parametric *t*-test) was performed using SPSS 18.0 software (IBM Co., Armonk, NY, United States). Differences are considered significant with ^∗^*P* < 0.05 or *^∗∗∗^P* < 0.001.

## Results and Discussion

### Transcriptomic Changes of *S. mutans* within Mixed-Species Biofilms

**FIGURE 1:**
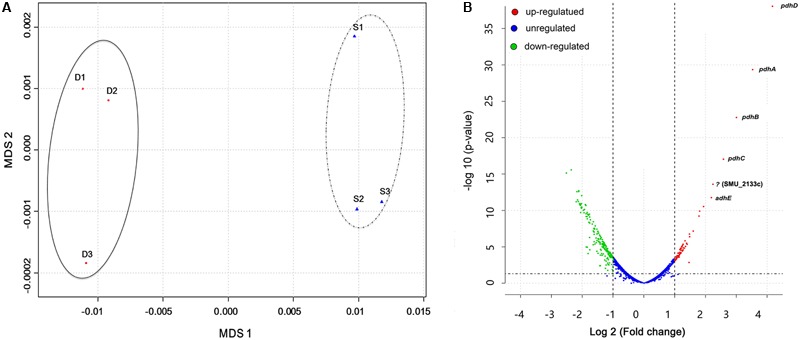
Overall transcriptomic changes of *S. mutans* within *S. mutans–C. albicans* dual biofilm. **(A)** Multi-Dimensional Scaling (MDS) plot based on Euclidean distances derived from a sample variance parameter, showing the level of correlation and reproducibility among samples. Red circles (D) show dual biofilm, whereas blue triangles (S) show single. Samples from single and dual biofilm group clustered together, respectively, indicating the different transcriptome pattern of *S. mutans* with and without *C. albicans*. **(B)** Plot showing fold change and levels of significance for differential expression for all genes.

Results from previous studies have shown enhanced *S. mutans* growth and biofilm formation as well as alterations in gene expression when co-cultured with *C. albicans* in the presence of sucrose (Falsetta et al., 2014; Sztajer et al., 2014). However, whole bacterial transcriptome characterization of effects of the presence of *C. albicans* in the dual-species biofilm milieu in combination with gene network pathway analysis is needed to gain a comprehensive picture of the underlying molecular mechanisms. To achieve this, we used our extensively optimized RNA extraction and mRNA enrichment protocol. Total RNA was isolated either from *S. mutans* single-species or *S. mutans*–*C. albicans* co-cultivation biofilms, and prokaryotic mRNA was enriched as described above and in the Supplementary Figure S1. A total of 587909293 reads were produced from the six samples. The sequence reads of all samples were deposited in the NCBI sequence read archive (SRA) as a study under the accession number of (SRR5116699-5116704). We first applied Multi-Dimensional Scaling (MDS) to provide a visual representation of the transcriptomic similarities between dual- and single-species biofilms. Samples marked with distinct colors were projected to a two-dimensional space and clustered separately (**Figure 1A**), indicating high levels of correlation and reproducibility among samples, as well as distinctive transcriptome profiles from *S. mutans* in the presence or absence of *C. albicans.* Three statistical methods, including DEseq, edgeR, and limma, were used to pinpoint differentially expressed genes (DEGs) between groups.

Overall, 393 genes showed significant differences in expression between single and dual-species biofilm for all three statistical methods with log_2_ (fold change) > 0.6 or < -0.7 (Supplementary Table S1), accounting for ∼20% (393/2042) of the total genes annotated in *S. mutans* UA159. Among the DEGs, 134 were up-regulated, 259 were down-regulated, and about 40% (158/393 genes) were of unknown function or hypothetical. The genes encoding the four-enzyme pyruvate dehydrogenase complex [*pdhD* (SMU_1424)-*pdhA* (SMU_1423)*-pdhB* (SMU_1422)*-pdhC* (SMU_1421)] and *adhE* (SMU_148) showed the highest up-regulation in mixed-species biofilms (fold change > 4.5, **Figure 1B**). All of these five genes are part of the pyruvate metabolism pathway, converting pyruvate to acetyl-CoA in cells growing in aerobic conditions. The gene (SMU_2133c) marked with a question mark in **Figure 1B** is not part of the pyruvate pathway and has an ambiguous annotation: hypothetical protein, transmembrane protein, or phage infection protein. Based on the RNA-seq data, we selected 10 DEGs (three down-regulated and seven up-regulated) showing a broad range of differential expression for validation by using qRT-PCR analysis. Consistent with the RNA-Seq data, the qPCR data showed significant differential expression of all genes tested (**Table 1**) and a linear-correlation with RNA-seq data (*r*^2^ = 0.98). We noted that the level of differential expression of some genes (e.g., *gtfB, gtfC*) was not entirely similar to that reported in our previous work (Falsetta et al., 2014). Differences between the two studies may be due to several factors, including different RNA sources/preparation (total RNA vs. rRNA depleted/mRNA enriched) and algorithm/data analysis to calculate fold changes. Despite differences in the level of gene expression, both studies confirmed up-regulation of *gtfBC* in dual-species biofilm (vs. single-species *S. mutans* biofilm).

**Table 1:** Validation of RNA-Seq data by qPCR.

Gene	Fold change (dual/single)	
	RNA-seq	qPCR^a^ (normalized by *gyrA*)
*comC*	0.46	0.30 ± 0.03
*luxS*	0.60	0.60 ± 0.04
*atpB*	0.62	0.64 ± 0.03
*hrcA*	1.57	1.57 ± 0.09
*SMU.104*	1.80	1.77 ± 0.14
*ciaR*	2.04	2.06 ± 0.21
*lacC*	2.25	1.83 ± 0.09
*gbpC*	2.26	2.36 ± 0.16
*adhE*	4.49	3.90 ± 0.14
*pdhA*	11.39	8.12 ± 1.60

*^a^Changes in transcript levels were determined using *gyr*A as an internal control and qRT-PCR results are presented as averages ± standard deviations.*

**FIGURE 2:**
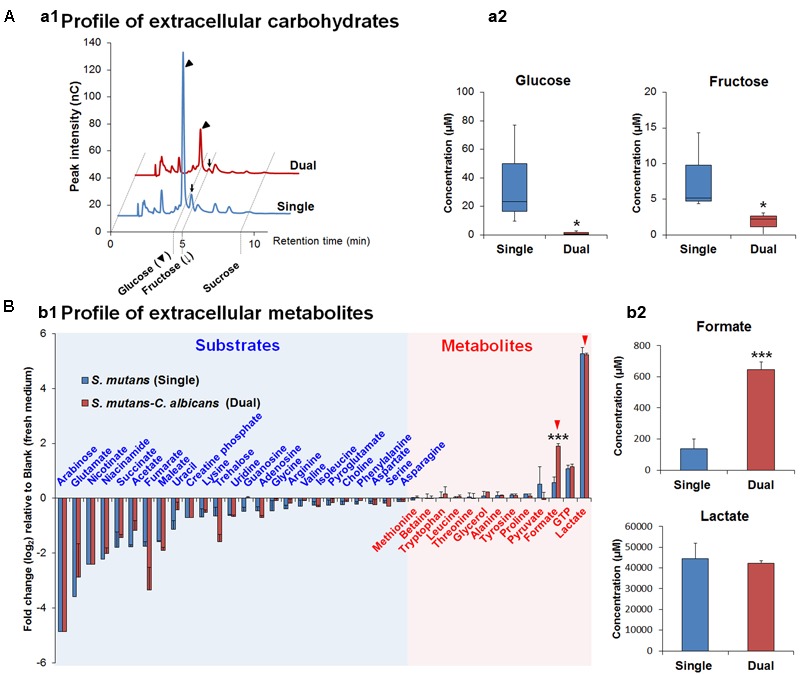
The composition of extracellular carbohydrates and metabolites. **(A)** HPAEC chromatograms of selected carbohydrates profile **(a1)** and the concentrations of glucose and fructose as the main carbohydrates (sucrose is not detected in both supernatants) in biofilm-culture supernatants **(a2)**. In the box whisker plots, whiskers represent minimum and maximum, and the box represents the 25th and 75th percentiles (*n* = 3). ^∗^*P* < 0.05. **(B)** Profiles of extracellular metabolites (e.g., organic acids, alcohols, sugar alcohols, amino acids) **(b1)** and the concentrations of formate and lactate (red arrowheads) as the main organic acids, which is associated with cariogenic properties of *S. mutans*, in biofilm-culture supernatants **(b2)**. Substrates and metabolites are characterized by the calculation of fold changes (log_2_) relative to the blank (fresh UFTYE medium). Data represent mean ± standard deviations (*n* = 3). ^∗∗∗^*P* < 0.001.

Gene Ontology terms were assigned to all genes in the *S. mutans* genome. We compared terms for the up- and down-regulated genes to a background of all terms to obtain an overall insight into the impact of *C. albicans* on *S. mutans* when growing together in dual-species biofilms. Forty-two GO terms were overrepresented (enriched) (Supplementary Table S2): among them, 22 were involved with biological processes, of which 10 with up-regulated genes and 12 with down-regulated genes (**Table 2**). Notably, all the GO terms for up-regulated genes belonged to the biological process domain and were involved in carbohydrate transport and metabolic/catabolic process. These findings are interesting since sugar catabolism is a key risk factor for dental caries (Selwitz et al., 2007), and are consistent with enhanced sugar utilization in dual-species biofilm (vs. single-species *S. mutans* biofilm) as determined by chromatographic analyses (**Figure 2A**). Since *Candida* is rather inefficient in metabolizing sucrose (Williamson et al., 1993), *S. mutans* can cross-feed sucrose break-down products (glucose and fructose) to *C. albicans* (Sztajer et al., 2014; Kim et al., 2017). We observed that the concentrations of glucose and fructose in the supernatant of dual-species biofilm are significantly lower than those in single-species *S. mutans* biofilm (**Figure 2A-a2**; *P* < 0.05). The data indicate that *S. mutans* co-cultured with *C. albicans* utilized most of the fermentable sugars, while also increasing the levels of formate (**Figure 2B**). Hence, once they are together within biofilm, these organisms may cooperate with each other for provision of sugar substrates and metabolites. Conversely, enhanced sugar utilization can also cause localized carbohydrate limitation in the presence of *Candida* that could influence the gene expression profile and the bacterial metabolic pathways.

**Table 2:** GO terms for biological processes with up and down-regulated genes for *S. mutans* grown with *C. albicans.*

Up regulated biological process	Down regulated biological process
Disaccharide metabolic process	Translation
Oligosaccharide metabolic process	Multi-organism process
Cellular carbohydrate catabolic process	Cellular protein metabolic process
Oligosaccharide catabolic process	Response to external biotic stimulus
Disaccharide catabolic process	Response to other organism
Phosphoenolpyruvate-dependent sugar	Response to biotic stimulus
phosphotransferase system	Defense response to other organism
Lactose metabolic process	Response to external stimulus
Carbohydrate metabolic process	Defense response
Carbohydrate transport	Protein metabolic process
	Defense response to bacterium
	Response to bacterium

**FIGURE 3:**
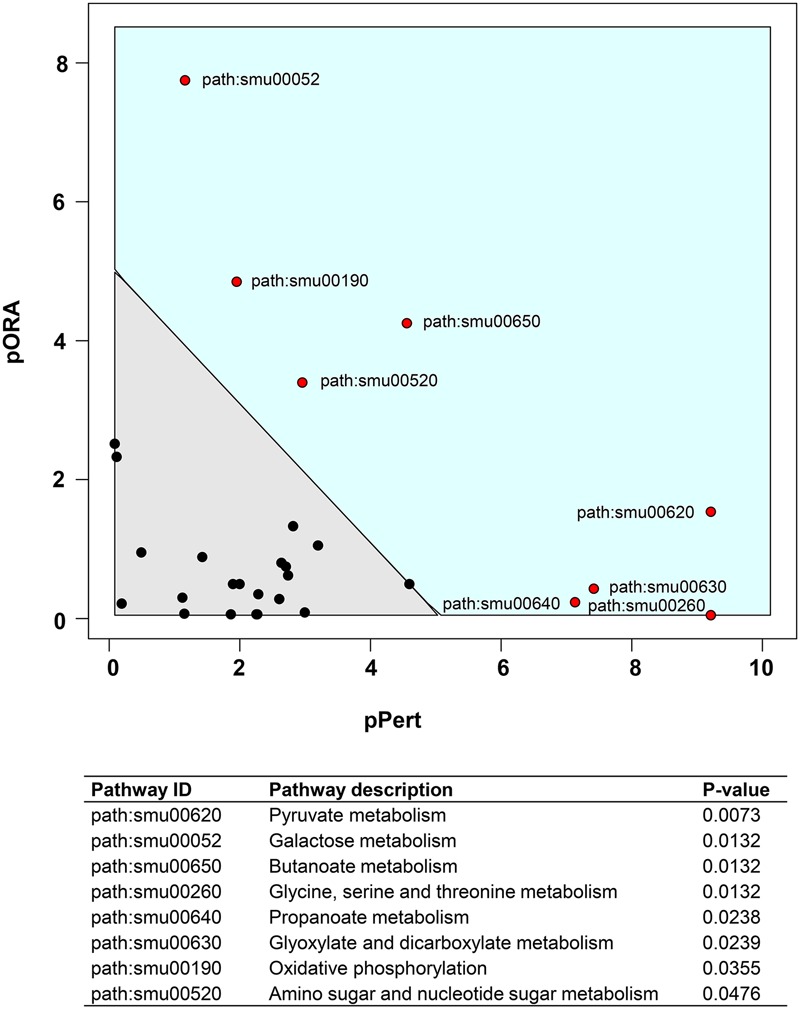
Plot showing result of a pathway impact analysis as implemented in Pathway-Express. The y-axis shows evidence for over-representation of differentially expressed genes in a pathway and the *x*-axis shows perturbation evidence (measured expression changes propagating across the pathway topology). A combination of the factors on each axis equates to the level of impact and the diagonal line represents a significance threshold (α = 0.05). After FDR correction, eight KEGG pathways (red dots) remained significant. Two pathways (pyruvate and galactose metabolism) at the extremes of the axes showed the most impact.

We also performed a KEGG pathway impact analysis based on the sequencing data. The analysis detected eight KEGG pathways that were significantly impacted: pyruvate metabolism, galactose metabolism, butanoate metabolism, glycine, serine and threonine metabolism, propanoate metabolism, glyoxylate and dicarboxylate metabolism, oxidative phosphorylation, and amino sugar and nucleotide sugar metabolism (**Figure 3** and Supplementary Table S3). Highly consistent with GO analysis, most of the impacted pathways were involved in carbohydrates metabolism. The end products of sugar fermentation are energy generation and predominantly organic acids, which can provide advantages for *S. mutans* survival and growth, while acidification of the environment helps both *S. mutans* and *C. albicans* (highly acid-tolerant organisms) to outcompete beneficial commensal bacteria (Burne and Marquis, 2001; Klinke et al., 2009). This metabolic cooperation provides an effective mechanism that promotes co-existence while enhancing *S. mutans* accumulation (Falsetta et al., 2014; Kim et al., 2017). At the same time, the bacterial population is probably heterogeneous with respect to carbohydrate utilization, and the transcriptome pattern could be compartmentalized within the biofilm. It is apparent that when *S. mutans* and *C. albicans* are together within biofilms when conditions are conducive for ECC, the presence of *Candida* modulates carbohydrate utilization while also creating carbohydrate-limiting conditions, both of which can activate the PDH pathway.

### Co-culturing with *C. albicans* Modulates Carbohydrate Utilization by *S. mutans*

**FIGURE 4:**
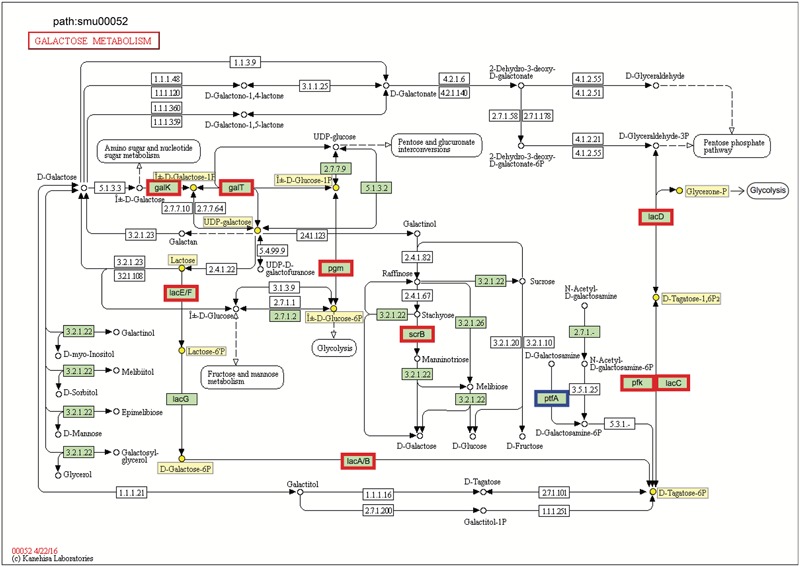
KEGG galactose metabolism pathway map (smu:00052) for *S. mutans* UA159. *S. mutans* genes involved in the pathway are shown in green. Of these, eleven showed differential expression. The nine bordered in red were up-regulated and the one bordered in blue was down-regulated. Six of the seven genes comprising the galactose pathway for lactose catabolism (*lac*E, *lac*F, *lac*G, *lac*A, *lac*B, *lac*C, and *lac*D) were up-regulated, and two genes of Leloir pathway (*gal*K and *galT*) were up-regulated. Numbers inside boxes are enzyme commission numbers.

The increased sugar metabolism can explain in part the increased carriage of *S. mutans* and *C. albicans* and enhanced virulence of plaque-biofilms *in vivo* (Falsetta et al., 2014). Sucrose, in particular, has long been considered the most cariogenic of all carbohydrates. This disaccharide serves as both a readily metabolizable carbon and energy source and as an essential substrate for the synthesis of the adhesive extracellular glucan matrix by *S. mutans*, which strengthens the interactions between *S. mutans* and *C. albicans* and augments the stability of biofilms containing these organisms (Gregoire et al., 2011; Falsetta et al., 2014). In addition to sucrose utilization extracellularly (Bowen and Koo, 2011), *S. mutans* rapidly transports sucrose into the cell by the phosphoenolpyruvate:sugar phosphotransferase system (PTS) (Ajdić and Pham, 2007; Moye et al., 2014). Here, we observed that *scrA* (SMU_1841), *scrB* (SMU_1843), and *scrK* (SMU_1840) were significantly up-regulated in dual-species biofilms (vs. single species *S. mutans*; Supplementary Table S1). The *scr*A gene encodes a high-affinity sucrose PTS permease, EII^Scr^, that internalizes sucrose as sucrose-6-phosphate (Sato et al., 1989). The ScrB enzyme is a sucrose-6-PO_4_ hydrolase that produces glucose-6-PO_4_ and fructose (**Figure 4**). After phosphorylation of fructose to fructose-6-phosphate by a fructokinase (*scrK*), the phosphorylated products are channeled into the glycolytic pathway (Chassy and Victoria Porter, 1979). Furthermore, *pttB* (SMU_2038), encoding a trehalose PTS permease, was also up-regulated in dual-species biofilms (Supplementary Table S1). Notably, the trehalose-PTS (i.e., EII^Tre^), the primary transporter for trehalose (Poy and Jacobson, 1990), is also able to transport sucrose, as mutants derived from *S. mutans* UA159 that lacked ScrA could still internalize sucrose via the PTS if an intact EII^Tre^ was present (Zeng and Burne, 2013). Besides the PTS, the multiple-sugar metabolism system (Msm) (Tao et al., 1993) and the maltose/maltodextrin ABC transporter (Kilic et al., 2007) have been also implicated in sucrose uptake by *S. mutans*, albeit not nearly as effectively as the sucrose PTS. Still, both *malG* (SMU_1570) and *malF* (SMU_1569) encoding maltose/maltodextrin ABC transport permease, as well as *malX* (SMU_1568) encoding maltose/maltodextrin-binding protein were up-regulated in the presence of *C. albicans* (Supplementary Table S1).

Genes for galactose metabolism by *S. mutans* were also up-regulated in the presence of *C. albicans*, which could simply reflect relief of catabolite repression. Another possible explanation is that *C. albicans* can produce galactose via up-regulation of its metabolic pathway by *N*-acetylglucosamine (GlcNAc) (Kamthan et al., 2013), which is a ubiquitous dietary sugar and also produced through bacterial biosynthesis (Moye et al., 2014). We found detectable amounts of GlcNAc in the supernatant of biofilm cultures of *S. mutans–C. albicans* [∼20 μM; compared to *S. mutans* alone (∼10 μM) (data not shown)], which may help to explain, at least in part this observation. *S. mutans* can metabolize galactose by two distinct pathways: the tagatose 6-phosphate pathway (de Vos and Vaughan, 1994) and Leloir pathway (Ajdić et al., 1996). Previous studies have shown that *S. mutans* can efficiently metabolize when both the tagatose 6-phosphate pathway and Leloir pathways are functional, while the tagatose pathway is responsible predominantly for the utilization of the phosphorylated galactose moiety that comes from the breakdown of lactose 6-phosphate (Abranches et al., 2004; Zeng et al., 2010).

In *S. mutans*, the genes encoding the tagatose 6-phosphate pathway are arranged as part of the *lac* operon. When co-culturing with *C. albicans*, six of the seven genes comprising tagatose 6-phosphate pathway [*lacE* (SMU_1491), *lacF* (SMU_1492), *lacA* (SMU_1496), *lacB* (SMU_1495), *lacC* (SMU_1494), and *lacD* (SMU_1493)] were up-regulated (**Figure 4** and Supplementary Table S4), which are consistent with their sequential role in the galactose metabolism. The galactose moiety of lactose, and possibly galactose alone, can be transported and phosphorylated by a lactose-specific (LacEF) PTS. The resultant galactose 6-phosphate generated by an intracellular 6-phospho-β-galactosidase is converted into tagatose 6-phosphate, then to tagatose-1,6-bisphosphate, and then to glyceraldehyde 3-phosphate and dihydroxyacetone by the enzymes galactose-6-phosphate isomerase (*lacAB*), tagatose 6-phosphate kinase (*lacC*), and tagatose-1,6-bisphosphate aldolase (*lacD*), respectively (Abranches et al., 2004). Furthermore, two genes of Leloir pathway [*galK* (SMU_886) and *galT* (SMU_887)] were also up-regulated (**Figure 4**), although the GalK pathway is minor and not as efficient as the tagatose pathway in *S. mutans* (Abranches et al., 2004). In the Leloir pathway, galactose enters the cell via an unidentified permease, where it is phosphorylated by galactokinase (*galK*) to yield galactose 1-phosphate, which is then converted into glucose 1-phosphate by hexose1-phosphate uridyltransferase (*galT*) and UDP-glucose epimerase [*galE* (SMU_888)]. The resulting glucose 1-phosphate can enter the glycolytic pathway.

### Pyruvate Metabolism of *S. mutans* in Dual-Species Biofilms

**FIGURE 5:**
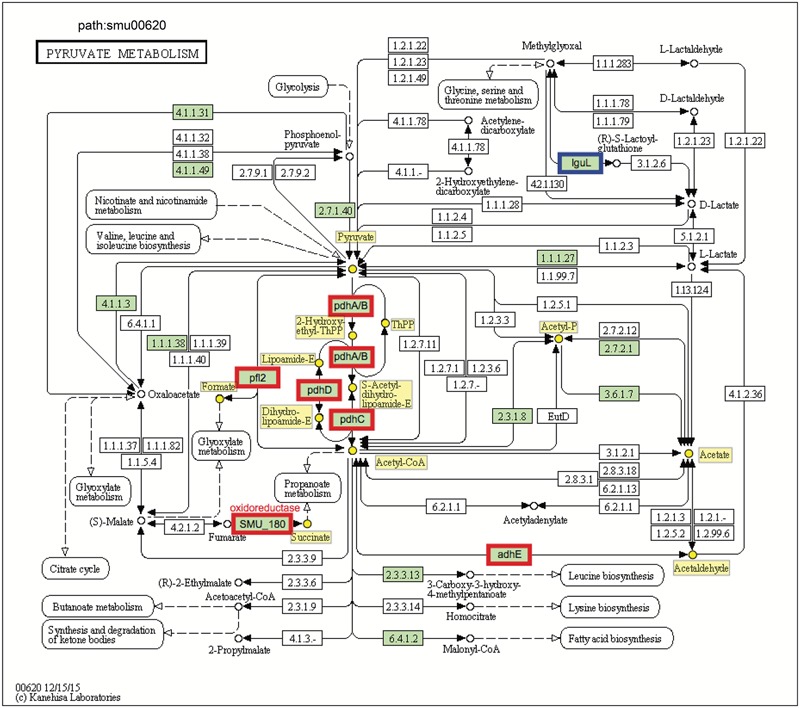
KEGG pyruvate metabolism pathway map (smu:00620) for *S. mutans* UA159. *S. mutans* genes involved in the pathway are shown in green. Of these, eight showed differential expression. The six bordered in red were up-regulated and the one bordered in blue was down-regulated. At the center of the pathway, showing strong up-regulation is the four-enzyme pyruvate dehydrogenase complex (*pdhD–pdhA–pdhB–pdhC*) (PDH).

The transcriptomic data suggest that *C. albicans* promotes *S. mutans* sugar utilization, leading to induction and/or derepression of genes for multiple catabolic pathways via inputs from global and specific regulatory systems. Among them, pyruvate metabolism is an important mechanism for *S. mutans* survival and expression of virulence within cariogenic biofilms that balances the need for ATP with maintenance of NAD/NADH ratios and for carbon for amino acid biosynthesis (Kim et al., 2015). Our pathway impact analysis shows that the pyruvate metabolism is substantially altered when *S. mutans* is growing in the presence of *C. albicans*, compared to single-species biofilms of *S. mutans* (**Figures 1B, 5** and Supplementary Table S4). All the genes in the *pdh* operon (*pdhD–pdhA–pdhB–pdhC*), as well as *pfl*2 (SMU_493) encoding PFL (**Figure 5**) and *pfl*A (SMU_1692) encoding PFL activating enzyme (Supplementary Table S4) were up-regulated in dual-species biofilm (vs. single-species *S. mutans*). It is conceivable that pyruvate metabolism was different between single- and dual-biofilms since both the glucose and fructose from sucrose and galactose catabolism lead to pyruvate production.

Pyruvate sits at an intersection of key pathways of sugar metabolism, and is converted by (1) pyruvate dehydrogenase complex (PDHc) into acetyl-CoA and CO_2_, (2) pyruvate formate lyase (PFL) yielding acetyl-CoA and formate, and (3) lactate dehydrogenase (LDH) into lactate that can be further metabolized. PDH and PFL pathways are activated when carbohydrates are not present in excess, suggesting that at least some cells in the dual-species biofilm might experience limitation for carbohydrate (**Figure 2**). Both GO and KEGG results indicate *S. mutans* and *C. albicans* may be competing for the fermentable sugar available in the culture medium when grown together in biofilms, which could trigger derepression or activation of alternative transport and catabolic pathways of *S. mutans*, as well as other adaptive mechanisms in response to environmental changes that enhance carbohydrate utilization (vs. single-species *S. mutans* biofilm). Such a scenario would also be consistent with activation of the PDH pathway, since *S. mutans* growing with excess carbohydrate predominantly shunt carbon through LDH. Notably, the inactivation of *pdh* impairs the survival of *S. mutans* in limiting sugar conditions in stationary phase (Busuioc et al., 2010), such that activation of *pdh* could be important for survival or persistence of *S. mutans* or a sub-population of *S. mutans* in the mixed-culture system employed here.

In addition to producing energy for growth and anabolic processes, the *pdh* operon has also been shown to be vital for survival of sugar-starved *S. mutans* (Busuioc et al., 2010) and its acid tolerance (Korithoski et al., 2008), which are critical virulence properties within cariogenic biofilms. When carbohydrates are in excess, the LDH enzyme is allosterically activated by fructose-1,6-biphosphate, a glycolytic intermediate, to catalyze the conversion of pyruvate to lactate via generating NAD^+^ from NADH (homolactic fermentation). Notably, LDH would be less active in carbohydrate-limiting conditions and utilization of galactose via the tagatose pathway (which is activated in dual-species) bypasses the production of the intermediates that regulate carbohydrate regulation (F-1,6-bP, G-6-P) (Zeng et al., 2010). However, in carbohydrate-liming conditions, the metabolic shift between pyruvate and formate is controlled by PFL, which can convert pyruvate to formate and acetyl-CoA via up-regulated pyruvate metabolism (heterolactic fermentation). Acetyl-CoA can be further converted to acetyl-phosphate (acetyl-P), which can be used for the production of ATP via acetate kinase. Importantly, PFL appears to have a key role in pyruvate metabolism of *S. mutans* residing within natural dental biofilm (Abbe et al., 1982).

We detected higher concentrations of extracellular formate in dual-species biofilms (∼five-fold increase vs. single-species) as determined via ^1^H-NMR (*P* < 0.001, **Figure 2B-b2**), which was consistent with the increased expression of the genes for PFL. In contrast, *ldh* expression between dual-species and single-species *S. mutans* biofilms was unaffected and similar amounts of lactate were found in the culture medium (**Figure 2B-b2**). Formate, a stronger acid (pKa = 3.75 vs. pKa of lactate = 3.86), has been detected in significant amounts in resting cariogenic plaque in humans (Distler and Kröncke, 1986). Thus, induction of *S. mutans* PDH and PFL by the fungal presence within biofilms may contribute to the enhanced cariogenicity observed *in vivo* (Falsetta et al., 2014). Enhanced sugar metabolism induced by co-culturing of *C. albicans* combined with transcriptomic changes in PDH and PFL may offer at least one explanation for the carbohydrate limitation (**Figure 2**) and the observed gene expression patterns in dual-species biofilms. Moreover, PFL is inactivated by oxygen, whereas PDH production is enhanced by growth in air. The up-regulation of PDH and PFL pathways also suggest the potential for complex and spatially heterogeneous gene expression patterns, whereby cells are carbohydrate-limited, but differences in exposure of cells within certain regions of the biofilm may influence whether the PFL pathway (anaerobic) is active or cells predominantly use PDH (aerobic) to favor acetate production and generation of additional ATP.

### *C. albicans* Can also Promote *S. mutans* Fitness and Virulence through Signal Transduction System

Two-component signal transduction systems (TCSTS) involve phosphotransfer events between transmembrane sensor kinases and cytoplasmic response regulators, which are transcription factors that bind DNA to repress and/or activate gene expression (Stock et al., 2000). Currently, 14 TCSTS have been identified that are able to enhance the ecological fitness and cariogenic potential of *S. mutans* (Smith and Spatafora, 2012). Genes encoding CiaRH [i.e., *ciaR* (SMU_1129) and *ciaH* (SMU_1128)] were up-regulated in the presence of *C. albicans* (two-fold), and this system has been implicated in acid tolerance, sucrose-dependent adherence and biofilm formation by *S. mutans* (Qi et al., 2004; Ahn et al., 2006; Biswas et al., 2008). Although in a different bacterial-fungal biofilm system, Dutton et al. (2016) also observed that *ciaR* gene in *S. gordonii* was up-regulated in early-stage of interaction with *C. albicans.* The gene *comC* (SMU_1915) encoding competence stimulating peptide (CSP), as well as several late competence genes, including *comYB* (SMU_1985), *comYD* (SMU_1983), *comEA* (SMU_625), and *comEC* (SMU_626) were down-regulated [log_2_ (fold change) < -0.7]. Factors regulating the development of genetic competence have been shown to influence acid tolerance, biofilm formation, eDNA release and stress tolerance in general. In parallel, a recent microfluidic study revealed that CSP signaling to induce competence is highly sensitive to pH and can be turned-off even in mildly acidic conditions (<pH 6.0, Son et al., 2015). Thus, biofilm microenvironmental changes may down-regulate genes involved with the development of genetic competence.

### Other Potential Interactions between *S. mutans* and *C. albicans*

Another notable difference between mono- and dual-species biofilms was the marked down-regulation of genes associated with the production of a suite of small antimicrobial peptides (bacteriocins) termed mutacins by *S. mutans* when co-cultured with *C. albicans* (**Table 2** and Supplementary Table S1). Since the expression of mutacin genes, which is regulated mainly by the CSP-ComDE system, is considered to be important in the competition with early colonizers, including *S. sanguinis* and *S. gordonii* (Merritt and Qi, 2012), a less robust activation of mutacin gene expression in *S. mutans* could alter the microbial composition of oral biofilms in a way that promotes a symbiotic relationship between *S. mutans* and *C. albicans*. Furthermore, previous studies reveal that deletion of genes [*nlmA* (SMU_150) and *nlmB* (SMU_151)] encoding mutacins IV and V in *S. mutans* results in lower antimicrobial activity against *S. pyogenes* (Hale et al., 2005; Hossain and Biswas, 2011), so decreased mutacin gene expression has indeed been correlated with lower mutacin biological activity.

We observed significant down-regulation of *comC*, the precursor for competence-stimulating peptide (CSP) that is a direct activator of mutacins via ComDE (van der Ploeg, 2005). It was previous shown that *C. albicans* activated *S. mutans comS* and *sigX* (Sztajer et al., 2014), so the down-regulation of *comC* might be perceived as inconsistent with this previous study. However, ComCDE do not directly activate *comS* or *sigX* (*comX*). Indeed, the observed decrease of *comC* (the CSP precursor structure gene) expression is completely consistent with the fact that SMU_1914c, SMU_299c, SMU_1889c, SMU_423, SMU_150, and SMU_151, which encode bacteriocins or products required for bacteriocin production, were also down-regulated. Bacteriocin production is dominantly regulated by CSP, which acts directly through the ComDE TCS to activate bacteriocin gene expression (van der Ploeg, 2005). Previous studies showed that CSP can regulate *C. albicans* growth and morphogenesis (Jarosz et al., 2009). It is possible that *Candida*, in addition to promoting an acidic microenvironment, can produce several proteases that may inhibit CSP signaling system, similar to the inhibition of *S. mutans* bacteriocin production by *S. gordonii* challisin (Wang and Kuramitsu, 2005). Hence, the presence of *C. albicans* and its effects on the biofilm milieu could subvert mutacin production to enhance its own persistence. Of note, the low pH created by the combination of *C. albicans* and *S. mutans* may diminish the need for *S. mutans* to produce bacteriocins that mainly target comparatively acid-sensitive commensal streptococci.

We also detected genes associated with clusters of regularly interspaced short palindromic repeats (CRISPRs, i.e., SMU_1760c, SMU_1761c, and SMU_1762c) of *S. mutans* that were down-regulated in dual-species biofilms. Lack of CRISPR activity could be a direct reflection of decreased competence observed in mixed-species biofilms, which would decrease the amount of DNA being internalized by *S. mutans*. CRISPR are involved primarily in antiviral defenses in prokaryotes (Brouns et al., 2008). However, the role of *S. mutans* CRISPR in caries development or in *C. albicans*–*S. mutans* interactions is presently unknown, but may warrant further investigation if the change in CRISPR expression does reflect some response to the presence of fungi.

**FIGURE 6:**
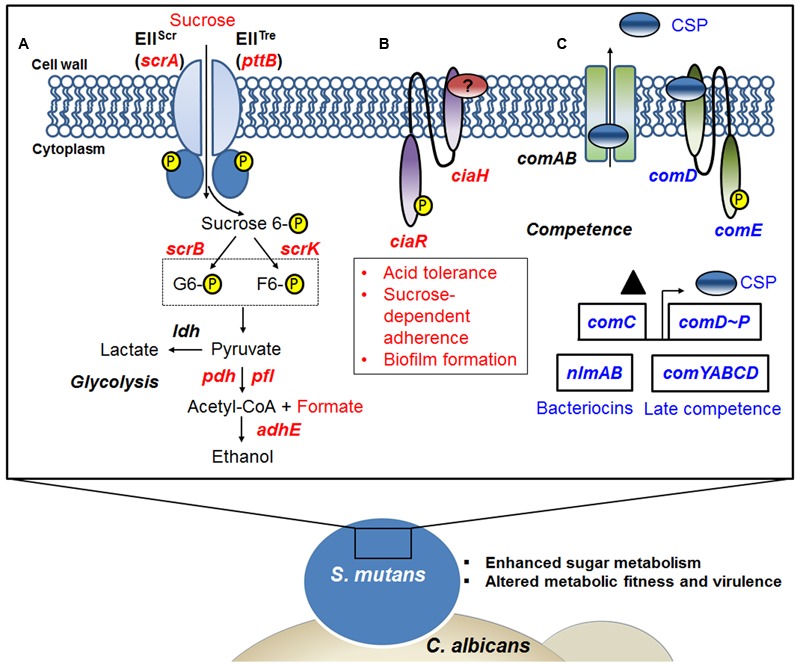
A snapshot of some of the transcriptome changes in *S. mutans* when co-cultured with *C. albicans*. Overall, the data reveal that the presence of *C. albicans* enhances sugar metabolism and metabolic fitness, and alters competence gene expression in *S. mutans*. **(A)** Up-regulation of the *scrA* gene encoding a high-affinity sucrose PTS permease (EII^Scr^) and possibly in concert with the product of the *pttB* gene encoding the trehalose-PTS (EII^Tre^) facilitates internalization of sucrose as sucrose-6-phosphate, with the glucose moiety carrying the phosphate group. Glucose-6-phosphate and fructose are produced via the action of ScrB, and ScrK used ATP to convert the fructose to fructose-6-phosphate. Both of these phosphohexoses can enter the glycolytic pathway. When *S. mutans* is growing in the presence of *C. albicans*, pyruvate metabolism can be affected by the availability of glycolytic intermediates. **(B)** The presence of *C. albicans* also appears to up-regulate the production of the CiaRH two component signal transduction system (TCSTS), which modulates acid tolerance, sucrose-dependent adherence and biofilm formation in *S. mutans*. **(C)** Another signaling system in *S. mutans*, the CSP-ComDE pathway, is affected by *C. albicans*. ComC (▲) is secreted and processed by ComAB to produce CSP (see text for more detail) and this peptide is then detected by the ComDE TCSTS. ComE directly activates bacteriocin production and CSP indirectly stimulates the development of genetic competence, as well as influencing cell. Indeed, changes of CSP-comDE pathway via down-regulation of *comC* can lead to down-regulation of downstream bacteriocin genes (e.g., *nlmAB*) and decreases in expression of late competence genes (*comYABCD*). Red letters indicate up-regulated genes, blue letters show down-regulated genes.

In summary, the present study provides a comprehensive insight into *S. mutans* transcriptomic changes associated with the presence of *C. albicans* within mixed-species biofilm. GO term and KEGG pathway impact analysis support an active influence on *S. mutans* at the transcriptional level. The fungal presence modulates the expression of genes involved in *S. mutans* sugar metabolism, fitness and survival within biofilms, offering plausible explanations for the enhanced bacterial accumulation and virulence of the bacterial-fungal biofilms in the context of ECC (**Figure 6**). RNA-Seq provides a detailed ‘snapshot’ of the overall transcriptome changes, but there are limitations. For example, RNA-Seq provide an average gene expression profile at a given time-point without taking into consideration the substantial spatio-temporal heterogeneity that exists within complex biofilms. Nevertheless, future studies using specific mutants of *S. mutans* can now be designed based on the RNA-Seq data with the goal of comparing the behaviors of the strains to interact and persist in co-culture system. This will include testing different mutant strains with altered abilities in carbohydrate metabolism and/or respond to changes in the environment caused by *C. albicans* presence, including CcpA as well as mutacin production (*nlmAB*) and cell–cell communication (*comDE*). Furthermore, future studies using recently developed single-cell *in situ* RNA-Seq (Lovatt et al., 2014) and a bacterial–fungal nanoculture system (Kim et al., 2017) for localized gene expression may facilitate description of the spatio-temporal transcriptome patterns within biofilms.

Although we focus on the influence of the presence of *C. albicans* on *S. mutans* transcriptome, *S. mutans* also provides benefits to *C. albicans*, such as enhanced colonization on the tooth surface and cross-feeding sucrose break-down products (e.g., glucose) for fungal utilization. Conversely, *S. mutans* could also impact *C. albicans* transcriptome based on recent observations that *S. gordonii* activate fungal genes associated with filamentation and proteases (Dutton et al., 2016). We are optimizing a protocol for both bacterial and fungal mRNA enrichment from mixed-species biofilms for dual RNA-Seq studies, which combined with metaproteomics, may provide additional mechanistic explanations. Clearly, this bacterium–fungus interaction is complex and multifaceted, and could induce additional cross-kingdom responses and alter the surrounding biofilm microenvironment to modulate the cariogenic potential of biofilms. We are currently exploring how the fungal infection is acquired and how *Candida* responds to the presence of *S. mutans* in cariogenic biofilms. Enhanced colonization and increased carriage of *C. albicans* in plaque biofilms may also provide a fungal reservoir that could promote *Candida* infections of oral mucosal surfaces. Thus, inclusion of antifungals may be an important factor for devising more effective therapies to control ECC and its consequences.

## Author Contributions

JH, DK, S-JA, RB, VR, and HK conceived the experiments; JH, DK, S-JA, and VR performed the experiments; JH, DK, XZ, S-JA, RB, VR, and HK analyzed the results and data interpretation; JH, DK, XZ, S-JA, RB, VR, and HK drafted and co-wrote the paper; JH, DK, XZ, S-JA, RB, VR, and HK final approval of the version to be published; JH, DK, XZ, S-JA, RB, VR, and HK agreed to be accountable for all aspects of the work in ensuring that questions related to the accuracy or integrity of any part of the work are appropriately investigated and resolved.

## Conflict of Interest Statement

The authors declare that the research was conducted in the absence of any commercial or financial relationships that could be construed as a potential conflict of interest.

## References

[B1] Abbe K. Takahashi S. Yamada T. (1982). Involvement of oxygen-sensitive pyruvate formate-lyase in mixed-acid fermentation by *Streptococcus mutans* under strictly anaerobic conditions. *J. Bacteriol.* 152 175–182. 6811549 10.1128/jb.152.1.175-182.1982 PMC221389

[B2] Abranches J. Chen Y.-Y. M. Burne R. A. (2004). Galactose metabolism by *Streptococcus mutans*. *Appl. Environ. Microbiol.* 70 6047–6052. 10.1128/AEM.70.10.6047-6052.2004 15466549 PMC522122

[B3] Ahn S.-J. Wen Z. T. Burne R. A. (2006). Multilevel control of competence development and stress tolerance in *Streptococcus mutans* UA159. *Infect. Immun.* 74 1631–1642. 10.1128/IAI.74.3.1631-1642.2006 16495534 PMC1418624

[B4] Ajdić D. Pham V. T. T. (2007). Global transcriptional analysis of *Streptococcus mutans* sugar transporters using microarrays. *J. Bacteriol.* 189 5049–5059. 10.1128/JB.00338-07 17496079 PMC1951856

[B5] Ajdić D. Sutcliffe I. C. Russell R. R. B. Ferretti J. J. (1996). Organization and nucleotide sequence of the *Streptococcus mutans* galactose operon. *Gene* 180 137–144. 8973358 10.1016/s0378-1119(96)00434-9

[B6] Anders S. Huber W. (2010). Differential expression analysis for sequence count data. *Genome Biol.* 11:R106 10.1186/gb-2010-11-10-r106 PMC3218662 20979621

[B7] Anders S. Pyl P. T. Huber W. (2015). HTSeq-a Python framework to work with high-throughput sequencing data. *Bioinformatics* 31 166–169. 10.1093/bioinformatics/btu638 25260700 PMC4287950

[B8] Benjamini Y. Hochberg Y. (1995). Controlling the false discovery rate: a practical and powerful approach to multiple testing. *J. R. Stat. Soc. Series B* 57 289–300.

[B9] Berkowitz R. J. Turner J. Hughes C. (1984). Microbial characteristics of the human dental caries associated with prolonged bottle feeding. *Arch. Oral Biol.* 29 949–951. 6596042 10.1016/0003-9969(84)90097-9

[B10] Biswas I. Drake L. Erkina D. Biswas S. (2008). Involvement of sensor kinases in the stress tolerance response of *Streptococcus mutans*. *J. Bacteriol.* 190 68–77. 10.1128/JB.00990-07 17965153 PMC2223747

[B11] Blüthgen N. Brand K. Cajavec B. Swat M. Herzel H. Beule D. (2005). Biological profiling of gene groups utilizing gene ontology. *Genome Inform.* 16 106–115. 10.11234/gi1990.16.106 16362912

[B12] Bowen W. H. Koo H. (2011). Biology of *Streptococcus mutans*-derived glucosyltransferases: role in extracellular matrix formation of cariogenic biofilms. *Caries Res.* 45 69–86. 10.1159/000324598 PMC3068567 21346355

[B13] Branting C. Sund M. L. Linder L. E. (1989). The influence of *Streptococcus mutans* on adhesion of *Candida albicans* to acrylic surfaces in vitro. *Arch. Oral Biol.* 34 347–353. 10.1016/0003-9969(89)90108-8 2532001

[B14] Brouns S. J. J. Jore M. M. Lundgren M. Westra E. R. Slijkhuis R. J. H. Snijders A. P. L. (2008). Small CRISPR RNAs guide antiviral defense in prokaryotes. *Science* 321 960–964. 10.1126/science.1159689 18703739 PMC5898235

[B15] Burne R. A. Marquis R. E. (2001). Biofilm acid/base physiology and gene expression in oral bacteria. *Methods Enzymol.* 337 403–415. 10.1016/S0076-6879(01)37029-5 11398446

[B16] Bustin S. A. Benes V. Garson J. A. Hellemans J. Huggett J. Kubista M. (2009). The MIQE guidelines: minimum information for publication of quantitative real-time PCR experiments. *Clin. Chem.* 55 611–622. 10.1373/clinchem.2008.112797 19246619

[B17] Busuioc M. Buttaro B. A. Piggot P. J. (2010). The pdh operon is expressed in a subpopulation of stationary-phase bacteria and is important for survival of sugar-starved *Streptococcus mutans*. *J. Bacteriol.* 192 4395–4402. 10.1128/JB.00574-10 20581205 PMC2937364

[B18] Calin V. Draghici S. (2016). *ROntoTools: R Onto-Tools Suite. R Package Version 1.10.0*.

[B19] Chassy B. M. Victoria Porter E. (1979). Initial characterization of sucrose-6-phosphate hydrolase from *Streptococcus mutans* and its apparent identity with intracellular invertase. *Biochem. Biophys. Res. Commun.* 89 307–314. 10.1016/0006-291X(79)90979-3 224874

[B20] Croucher N. J. Thomson N. R. (2010). Studying bacterial transcriptomes using RNA-seq. *Curr. Opin. Microbiol.* 13 619–624. 10.1016/j.mib.2010.09.009 20888288 PMC3025319

[B21] Cury J. A. Koo H. (2007). Extraction and purification of total RNA from *Streptococcus mutans* biofilms. *Anal. Biochem.* 365 208–214. 10.1016/j.ab.2007.03.021 17475197

[B22] de Carvalho F. G. Silva D. S. Hebling J. Spolidorio L. C. Spolidorio D. M. (2006). Presence of mutans streptococci and *Candida* spp. in dental plaque/dentine of carious teeth and early childhood caries. *Arch. Oral Biol.* 51 1024–1028. 10.1016/j.archoralbio.2006.06.001 16890907

[B23] de Vos W. M. Vaughan E. E. (1994). Genetics of lactose utilization in lactic acid bacteria. *FEMS Microbiol. Rev.* 15 217–237. 7946468 10.1111/j.1574-6976.1994.tb00136.x

[B24] Diaz P. I. Xie Z. Sobue T. Thompson A. Biyikoglu B. Ricker A. (2012). Synergistic interaction between *Candida albicans* and commensal oral streptococci in a novel *in vitro* mucosal model. *Infect. Immun.* 80 620–632. 10.1128/IAI.05896-11 22104105 PMC3264323

[B25] Distler W. Kröncke A. (1986). Formic acid in human single-site resting plaque-quantitative and qualitative aspects. *Caries Res.* 20 1–6. 10.1159/000260913 3455883

[B26] Dutton L. C. Paszkiewicz K. H. Silverman R. J. Splatt P. R. Shaw S. Nobbs A. H. (2016). Transcriptional landscape of trans-kingdom communication between *Candida albicans* and *Streptococcus gordonii*. *Mol. Oral Microbiol.* 31 136–161. 10.1111/omi.12111 26042999 PMC4670286

[B27] Falsetta M. L. Klein M. I. Colonne P. M. Scott-Anne K. Gregoire S. Pai C. H. (2014). Symbiotic relationship between *Streptococcus mutans* and *Candida albicans* synergizes virulence of plaque biofilms in vivo. *Infect. Immun.* 82 1968–1981. 10.1128/IAI.00087-14 24566629 PMC3993459

[B28] Ghannoum M. A. Jurevic R. J. Mukherjee P. K. Cui F. Sikaroodi M. Naqvi A. (2010). Characterization of the oral fungal microbiome (mycobiome) in healthy individuals. *PLoS Pathog.* 6:e1000713 10.1371/journal.ppat.1000713 PMC2795202 20072605

[B29] Gotz S. Garcia-Gomez J. M. Terol J. Williams T. D. Nagaraj S. H. Nueda M. J. (2008). High-throughput functional annotation and data mining with the Blast2GO suite. *Nucleic Acids Res.* 36 3420–3435. 10.1093/nar/gkn176 18445632 PMC2425479

[B30] Gregoire S. Xiao J. Silva B. B. Gonzalez I. Agidi P. S. Klein M. I. (2011). Role of glucosyltransferase B in interactions of *Candida albicans* with *Streptococcus mutans* and with an experimental pellicle on hydroxyapatite surfaces. *Appl. Environ. Microbiol.* 77 6357–6367. 10.1128/AEM.05203-11 21803906 PMC3187131

[B31] Gross E. L. Beall C. J. Kutsch S. R. Firestone N. D. Leys E. J. Griffen A. L. (2012). Beyond *Streptococcus mutans*: dental caries onset linked to multiple species by 16S rRNA community analysis. *PLoS ONE* 7:e477722 10.1371/journal.pone.0047722 PMC3472979 23091642

[B32] Hajishengallis E. Parsaei Y. Klein M. I. Koo H. (2017). Advances in the microbial etiology and pathogenesis of early childhood caries. *Mol. Oral Microbiol.* 32 24–34. 10.1111/omi.12152 26714612 PMC4929038

[B33] Hale J. D. F. Ting Y.-T. Jack R. W. Tagg J. R. Heng N. C. K. (2005). Bacteriocin (mutacin) production by *Streptococcus mutans* genome sequence reference strain UA159: elucidation of the antimicrobial repertoire by genetic dissection. *Appl. Environ. Microbiol.* 71 7613–7617. 10.1128/AEM.71.11.7613-7617.2005 16269816 PMC1287737

[B34] Hall-Stoodley L. Costerton J. W. Stoodley P. (2004). Bacterial biofilms: from the natural environment to infectious diseases. *Nat. Rev. Microbiol.* 2 95–108. 15040259 10.1038/nrmicro821

[B35] Hossain M. S. Biswas I. (2011). Mutacins from *Streptococcus mutans* UA159 are active against multiple streptococcal species. *Appl. Environ. Microbiol.* 77 2428–2434. 10.1128/AEM.02320-10 21296932 PMC3067413

[B36] Hwang G. Marsh G. Gao L. Waugh R. Koo H. (2015). Binding force dynamics of *Streptococcus mutans*-glucosyltransferase B to *Candida albicans*. *J. Dent. Res.* 94 1310–1317. 10.1177/0022034515592859 26138722 PMC4547317

[B37] Jarosz L. M. Deng D. M. van der Mei H. C. Crielaard W. Krom B. P. (2009). *Streptococcus mutans* competence-stimulating peptide inhibits *Candida albicans* hypha formation. *Eukaryot. Cell* 8 1658–1664. 10.1128/EC.00070-09 19717744 PMC2772401

[B38] Jenkinson H. F. Douglas L. J. (2002). “Candida interactions with bacterial biofilms,” in *Polymicrobial Diseases* eds Brogden K. A. Guthmiller J. M. (Washington, DC: ASM Press) 357–373. 21735561

[B39] Jenkinson H. F. Lala H. C. Shepherd M. G. (1990). Coaggregation of *Streptococcus sanguis* and other streptococci with *Candida albicans*. *Infect. Immun.* 58 1429–1436. 2182544 10.1128/iai.58.5.1429-1436.1990 PMC258643

[B40] Kamthan M. Kamthan A. Ruhela D. Maiti P. Bhavesh N. S. Datta A. (2013). Upregulation of galactose metabolic pathway by N-acetylglucosamine induced endogenous synthesis of galactose in *Candida albicans*. *Fungal Genet. Biol.* 54 15–24. 10.1016/j.fgb.2013.02.006 23454545

[B41] Kassebaum N. J. Bernabë E. Dahiya M. Bhandari B. Murray C. J. Marcenes W. (2015). Global burden of untreated caries: a systematic review and metaregression. *J. Dent. Res.* 94 650–658. 10.1177/0022034515573272 25740856

[B42] Kilic A. O. Honeyman A. L. Tao L. (2007). Overlapping substrate specificity for sucrose and maltose of two binding protein-dependent sugar uptake systems in *Streptococcus mutans*. *FEMS Microbiol. Lett.* 266 218–223. 10.1111/j.1574-6968.2006.00522.x 17233733

[B43] Kim D. Sengupta A. Niepa T. H. R. Lee B. H. Weljie A. Freitas-Blanco V. S. (2017). *Candida albicans* stimulates *Streptococcus mutans* microcolony development via cross-kingdom biofilm-derived metabolites. *Sci. Rep.* 7:41332 10.1038/srep41332 PMC5278416 28134351

[B44] Kim J. N. Ahn S.-J. Burne R. A. (2015). Genetics and physiology of acetate metabolism by the Pta-Act pathway of *Streptococcus mutans*. *Appl. Environ. Microbiol.* 81 5015–5025. 10.1128/AEM.01160-15 25979891 PMC4495203

[B45] Klinke T. Kneist S. de Soet J. J. Kuhlisch E. Mauersberger S. Forster A. (2009). Acid production by oral strains of *Candida albicans* and lactobacilli. *Caries Res.* 43 83–91. 10.1159/000204911 19246906

[B46] Klinke T. Urban M. Luck C. Hannig C. Kuhn M. Kramer N. (2014). Changes in *Candida* spp., mutans streptococci and lactobacilli following treatment of early childhood caries: a 1-year follow-up. *Caries Res.* 48 24–31. 10.1159/000351673 24216710

[B47] Koo H. Falsetta M. L. Klein M. I. (2013). The exopolysaccharide matrix: a virulence determinant of cariogenic biofilm. *J. Dent. Res.* 92 1065–1073. 10.1177/0022034513504218 24045647 PMC3834652

[B48] Koo H. Xiao J. Klein M. I. Jeon J. G. (2010). Exopolysaccharides produced by *Streptococcus mutans* glucosyltransferases modulate the establishment of microcolonies within multispecies biofilms. *J. Bacteriol.* 192 3024–3032. 10.1128/JB.01649-09 20233920 PMC2901689

[B49] Korithoski B. Lévesque C. M. Cvitkovitch D. G. (2008). The involvement of the pyruvate dehydrogenase E1α subunit, in *Streptococcus mutans* acid tolerance. *FEMS Microbiol. Lett.* 289 13–19. 10.1111/j.1574-6968.2008.01351.x 19054088

[B50] Law C. W. Chen Y. Shi W. Smyth G. K. (2014). voom: precision weights unlock linear model analysis tools for RNA-seq read counts. *Genome Biol.* 15:R29 10.1186/gb-2014-15-2-r29 PMC4053721 24485249

[B51] Lemos J. A. Burne R. A. (2008). A model of efficiency: stress tolerance by *Streptococcus mutans*. *Microbiology* 154 3247–3255. 10.1099/mic.0.2008/023770-0 18957579 PMC2627771

[B52] Lovatt D. Ruble B. K. Lee J. Dueck H. Kim T. K. Fisher S. (2014). Transcriptome in vivo analysis (TIVA) of spatially defined single cells in live tissue. *Nat. Methods* 11 190–196. 10.1038/nmeth.2804 24412976 PMC3964595

[B53] Marsh P. D. Moter A. Devine D. A. (2011). Dental plaque biofilms: communities, conflict and control. *Periodontol. 2000* 55 16–35. 10.1111/j.1600-0757.2009.00339.x 21134226

[B54] Merritt J. Qi F. (2012). The mutacins of *Streptococcus mutans*: regulation and ecology. *Mol. Oral Microbiol.* 27 57–69. 10.1111/j.2041-1014.2011.00634.x 22394465 PMC3296966

[B55] Metwalli K. H. Khan S. A. Krom B. P. Jabra-Rizk M. A. (2013). *Streptococcus mutans, Candida albicans*, and the human mouth: a sticky situation. *PLoS Pathog.* 9:e1003616 10.1371/journal.ppat.1003616 PMC3798555 24146611

[B56] Moulos P. Hatzis P. (2015). Systematic integration of RNA-Seq statistical algorithms for accurate detection of differential gene expression patterns. *Nucleic Acids Res.* 43:e25 10.1093/nar/gku1273 PMC4344485 25452340

[B57] Moye Z. D. Zeng L. Burne R. V. (2014). Fueling the caries process: carbohydrate metabolism and gene regulation by *Streptococcus mutans*. *J. Oral Microbiol.* 6:24878 10.3402/jom.v6.24878 PMC4157138 25317251

[B58] Palmer C. A. Kent R. Jr. Loo C. Y. Hughes C. V. Stutius E. Pradhan N. (2010). Diet and caries-associated bacteria in severe early childhood caries. *J. Dent. Res.* 89 1224–1229. 10.1177/0022034510376543 20858780 PMC2954266

[B59] Parisotto T. M. Steiner-Oliveira C. Silva C. M. Rodrigues L. K. Nobre-dos-Santos M. (2010). Early childhood caries and mutans streptococci: a systematic review. *Oral Health Prev. Dent.* 8 59–70. 10.3290/j.ohpd.a18828 20480056

[B60] Poy F. Jacobson G. R. (1990). Evidence that a low-affinity sucrose phosphotransferase activity in *Streptococcus mutans* GS-5 is a high-affinity trehalose uptake system. *Infect. Immun.* 58 1479–1480. 2323827 10.1128/iai.58.5.1479-1480.1990 PMC258652

[B61] Qi F. Merritt J. Lux R. Shi W. (2004). Inactivation of the ciaH gene in *Streptococcus mutans* diminishes mutacin production and competence development, alters sucrose-dependent biofilm formation, and reduces stress tolerance. *Infect. Immun.* 72 4895–4899. 10.1128/IAI.72.8.4895-4899.2004 15271957 PMC470703

[B62] Qiu R. Li W. Lin Y. Yu D. Zhao W. (2015). Genotypic diversity and cariogenicity of *Candida albicans* from children with early childhood caries and caries-free children. *BMC Oral Health* 15:144 10.1186/s12903-015-0134-3 PMC4650516 26576955

[B63] Raja M. Hannan A. Ali K. (2010). Association of oral candidal carriage with dental caries in children. *Caries Res.* 44 272–276. 10.1159/000314675 20516688

[B64] Robinson M. D. McCarthy D. J. Smyth G. K. (2010). edgeR: a Bioconductor package for differential expression analysis of digital gene expression data. *Bioinformatics* 26 139–140. 10.1093/bioinformatics/btp616 19910308 PMC2796818

[B65] Rocha D. J. P. Santos C. S. Pacheco L. G. C. (2015). Bacterial reference genes for gene expression studies by RT-qPCR: survey and analysis. *Antonie Van Leeuwenhoek* 108 685–693. 10.1007/s10482-015-0524-1 26149127

[B66] Sato Y. Poy F. Jacobson G. R. Kuramitsu H. K. (1989). Characterization and sequence analysis of the scrA gene encoding enzyme IIScr of the *Streptococcus mutans* phosphoenolpyruvate-dependent sucrose phosphotransferase system. *J. Bacteriol.* 171 263–271. 2536656 10.1128/jb.171.1.263-271.1989 PMC209581

[B67] Selwitz R. H. Ismail A. I. Pitts N. B. (2007). Dental caries. *Lancet* 369 51–59. 10.1016/S0140-6736(07)60031-2 17208642

[B68] Smith E. G. Spatafora G. A. (2012). Gene Regulation in *S. mutans*: complex control in a complex environment. *J. Dent. Res.* 91 133–141. 10.1177/0022034511415415 21743034

[B69] Son M. Ghoreishi D. Ahn S.-J. Burne R. A. Hagen S. J. (2015). Sharply tuned pH response of genetic competence regulation in *Streptococcus mutans*: a microfluidic study of the environmental sensitivity of *comX*. *Appl. Environ. Microbiol.* 81 5622–5631. 10.1128/AEM.01421-15 26070670 PMC4510173

[B70] Stock A. M. Robinson V. L. Goudreau P. N. (2000). Two-component signal transduction. *Annu. Rev. Biochem.* 69 183–215. 10.1146/annurev.biochem.69.1.183 10966457

[B71] Sztajer H. Szafranski S. P. Tomasch J. Reck M. Nimtz M. Rohde M. (2014). Cross-feeding and interkingdom communication in dual-species biofilms of *Streptococcus mutans* and *Candida albicans*. *ISME J.* 8 2256–2271. 10.1038/ismej.2014.73 24824668 PMC4992082

[B72] Takahashi N. Nyvad B. (2011). The role of bacteria in the caries process: ecological perspectives. *J. Dent. Res.* 90 294–303. 10.1177/0022034510379602 20924061

[B73] Tanner A. C. Mathney J. M. Kent R. L. Chalmers N. I. Hughes C. V. Loo C. Y. (2011). Cultivable anaerobic microbiota of severe early childhood caries. *J. Clin. Microbiol.* 49 1464–1474. 10.1128/JCM.02427-10 21289150 PMC3122858

[B74] Tao L. Sutcliffe I. C. Russell R. R. B. Ferretti J. J. (1993). Transport of sugars, including sucrose, by the msm transport system of *Streptococcus mutans*. *J. Dent. Res.* 72 1386–1390. 8408880 10.1177/00220345930720100701

[B75] Thein Z. M. Seneviratne C. J. Samaranayake Y. H. Samaranayake L. P. (2009). Community lifestyle of *Candida* in mixed biofilms: a mini review. *Mycoses* 52 467–475. 10.1111/j.1439-0507.2009.01719.x 19486299

[B76] van der Ploeg J. R. (2005). Regulation of bacteriocin production in *Streptococcus mutans* by the quorum-sensing system required for development of genetic competence. *J. Bacteriol.* 187 3980–3989. 10.1128/JB.187.12.3980-3989.2005 15937160 PMC1151730

[B77] Wang B.-Y. Kuramitsu H. K. (2005). Interactions between oral bacteria: inhibition of *Streptococcus mutans* bacteriocin production by *Streptococcus gordonii*. *Appl. Environ. Microbiol.* 71 354–362. 10.1128/AEM.71.1.354-362.2005 15640209 PMC544254

[B78] Williamson P. R. Huber M. A. Bennett J. E. (1993). Role of maltase in the utilization of sucrose by *Candida albicans*. *Biochem. J.* 291 765–771. 10.1042/bj2910765 8489504 PMC1132434

[B79] Xu H. Sobue T. Thompson A. Xie Z. Poon K. Ricker A. (2014). Streptococcal co-infection augments *Candida* pathogenicity by amplifying the mucosal inflammatory response. *Cell. Microbiol.* 16 214–231. 10.1111/cmi.12216 24079976 PMC3956708

[B80] Yang X. Q. Zhang Q. Lu L. Y. Yang R. Liu Y. Zou J. (2012). Genotypic distribution of *Candida albicans* in dental biofilm of Chinese children associated with severe early childhood caries. *Arch. Oral Biol.* 57 1048–1053. 10.1016/j.archoralbio.2012.05.012 22717324

[B81] Zeng L. Burne R. A. (2013). Comprehensive mutational analysis of sucrose-metabolizing pathways in *Streptococcus mutans* reveals novel roles for the sucrose phosphotransferase system permease. *J. Bacteriol.* 195 833–843. 10.1128/JB.02042-12 23222725 PMC3562097

[B82] Zeng L. Burne R. A. (2016). Sucrose- and fructose-specific effects on the transcriptome of *Streptococcus mutans* probed by RNA sequencing. *Appl. Environ. Microbiol.* 82 146–156. 10.1128/AEM.02681-15 PMC4702655 26475108

[B83] Zeng L. Choi S. C. Danko C. G. Siepel A. Stanhope M. J. Burne R. A. (2013). Gene regulation by CcpA and catabolite repression explored by RNA-Seq in *Streptococcus mutans*. *PLoS ONE* 8:e60465 10.1371/journal.pone.0060465 PMC3610829 23555977

[B84] Zeng L. Das S. Burne R. A. (2010). Utilization of lactose and galactose by *Streptococcus mutans*: transport, toxicity, and carbon catabolite repression. *J. Bacteriol.* 192 2434–2444. 10.1128/JB.01624-09 20190045 PMC2863486

