# Supplementary material for: RNA-Seq Reveals Enhanced Sugar Metabolism in Streptococcus mutans Co-cultured with Candida albicans within Mixed-Species Biofilms
Source: Front Microbiol. 2017 Jun 8;8:1036. doi: 10.3389/fmicb.2017.01036 (PMC5462986; doi:10.3389/fmicb.2017.01036)
Supplement: Supplementary file 1 [file Data_Sheet_1.docx]

Supplementary Material

**RNA-Seq reveals enhanced sugar metabolism in *Streptococcus mutans* co-cultured with *Candida albicans* within mixed-species biofilms**

Jinzhi He^a,b,*^, Dongyeop Kim^b,*^, Xuedong Zhou^a^, Sang-Joon Ahn^c^, Robert A. Burne^c^, Vincent P. Richards^d^ , Hyun Koo^b#^

^a^State Key Laboratory of Oral Diseases, Department of Endodontics, West China Hospital of Stomatology, Sichuan University, Chengdu, China

^b^Biofilm Research Labs, Levy Center for Oral Health, Department of Orthodontics, School of Dental Medicine, University of Pennsylvania, Philadelphia, PA, USA

^c^Department of Oral Biology, College of Dentistry, University of Florida, Gainesville, FL, USA

^d^Department of Biological Sciences, Clemson University, Clemson, SC, USA

* These authors contribute equally as co-first author

**# Correspondence:** Hyun Koo: koohy@upenn.edu

## Supplementary Figure

# Figure S1

# Combined MICROBEnrich and Ribo-Zero approaches provide optimal bacterial mRNA enrichment from *C. albicans – S .mutans* biofilm.

The lack of polyadenylation at the 3’ end of bacterial mRNA, the abundance of rRNA (>95% of total RNA) and the presence of eukaryotic RNA provide unique challenges for prokaryotic gene expression profiling via RNA-Seq of complex biofilms. Although several approaches have been tested to eliminate rRNA or selectively transcribe mRNA using specific probes, there is no consensus in the available literature on the ideal methodology, and the published results are highly variable (He et al., 2010; Stewart et al., 2010; Giannoukos et al., 2012). Thus, we optimized two different approaches for bacterial mRNA enrichment using MICROBEnrich + MICROBExpress or MICROBEnrich + Ribo-Zero. Initially, total RNA was extracted and purified from *C. albicans* and *S. mutans* cells and mixed in equal amounts. The quality of the bacterial and fungal total RNA was analyzed via Agilent Pico-chip for RNA. As shown in Figure S1A, four peaks, corresponding to 16S, 18S, 23S and 28S rRNA, were clearly detected without degradation. Subsequently, the RNA samples were treated with MICROBEnrich. A single MICROBEnrich treatment efficiently removed the fungal RNA from the mixed total RNA, showing only 2 peaks (16S and 23S rRNA) (Figure S1B), while an additional treatment slightly improved enrichment without affecting the RNA yield (Figures S1B and S1C).

The RNA sample pre-treated with MICROBEnrich was then subjected to either MICROBExpress or Ribo-Zero treatment. We observed that a single MICROBExpress treatment was insufficient for bacterial rRNA depletion as evidenced by the presence of 23S rRNA peak (Figure S1D). However, a second treatment removed the majority of 23S rRNA (Figure 1E). In contrast, Ribo-Zero was capable of removing the fungal rRNA following a single treatment (Figure S1F), and the procedure was simpler than that of MICROBExpress. Based on these findings, we selected MICROBEnrich (twice) + Ribo-Zero (once) as an optimized method to enrich *S. mutans* mRNA from mixed bacterial-fungal RNA samples. Our results agree well with a previous study demonstrating that Ribo-Zero is an effective rRNA-depletion system (compared to MICROBExpress and RiboMinus) using a Gram negative bacterium *Salmonella enterica* SL1344 (Bhagwat et al., 2014). To further verify this enrichment protocol, we used total RNA isolated from both single and dual-species biofilms, and the results confirmed that the combination of MICROBEnrich and Ribo-Zero was effective at enriching prokaryotic mRNA from these samples (Figures S1G and S1H).


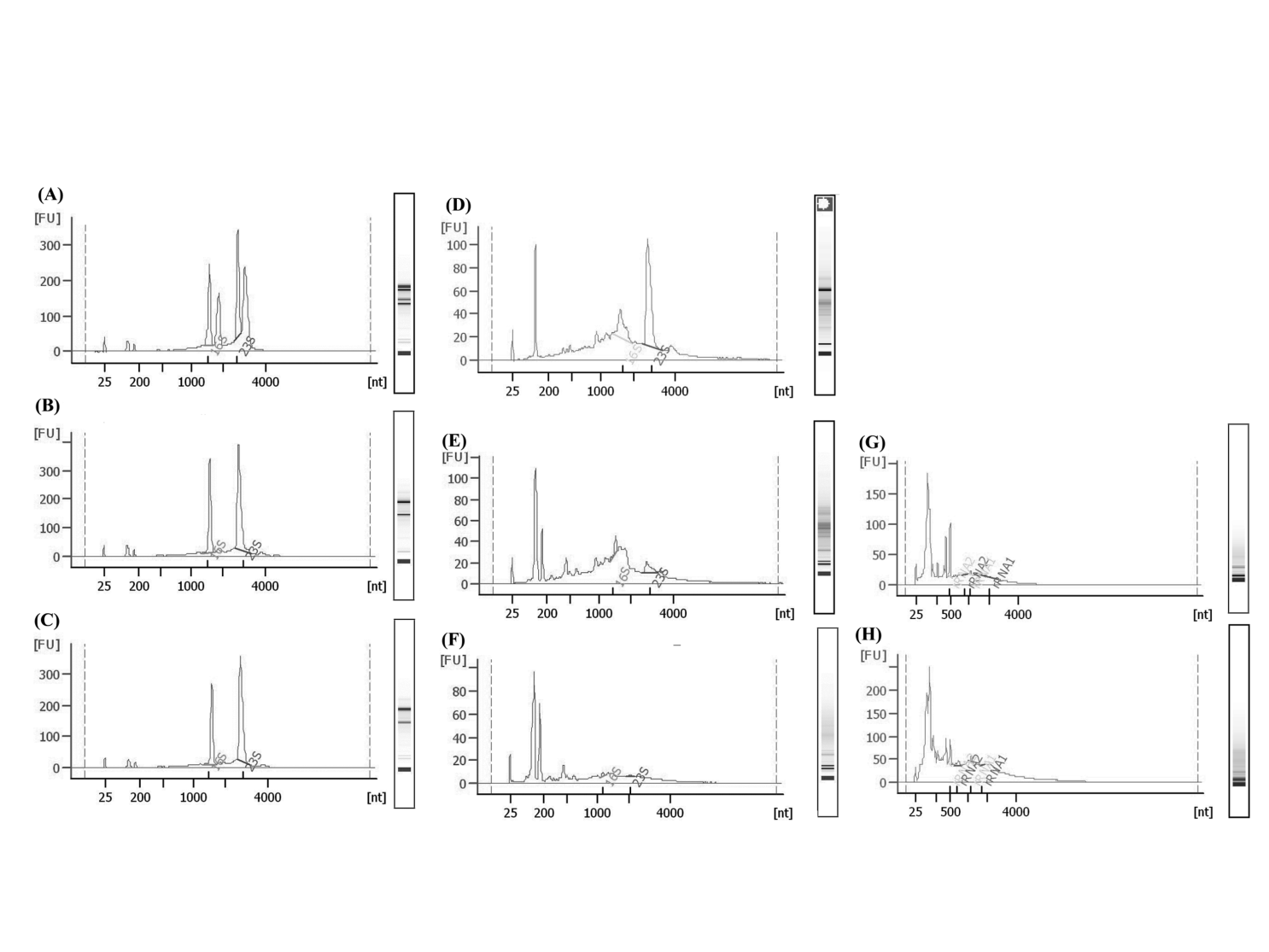
**Supplementary Figure S1. Comparison of different ways to enrich bacterial mRNA.** **(A)** Equal mixed *S.mutans* and *C. albicans* total RNA; **(B)** A + MICROBEnrich (once); **(C)** A+ MICROBEnrich (twice); **(D)** A + MICROBEnrich (twice) + MICROBExpress (once); **(E)** A + MICROBEnrich (twice) + MICROBExpress (twice); **(F)** A + MICROBEnrich (twice) + Ribo-zero; **(G)** Biofilm total RNA + MICROBEnrich (twice) + MICROBExpress (twice); **(H)** Biofilm total RNA + MICROBEnrich (twice) + Ribo-zero.

**References**

Giannoukos, G., Ciulla, D. M., Huang, K., Haas, B. J., Izard, J., Levin, J. Z., et al. (2012). Efficient and robust RNA-seq process for cultured bacteria and complex community transcriptome. *Genome Biol.* 13, 1–13. doi: 10.1186/gb-2012-13-3-r23

He, S., Wurtzel, O., Singh, K., Froula, J. L., Yilmaz, S., Tringe, S. G., et al. (2010). Validation of two ribosomal RNA removal methods for microbial metatranscriptomics. *Nat. Methods* 7, 807–812. doi: 10.1038/nmeth.1507

Stewart, F. J., Ottesen, E. A., and DeLong, E. F. (2010). Development and quantitative analyses of a universal rRNA-subtraction protocol for microbial metatranscriptomics. *ISME J.* 4, 896–907. doi: 10.1038/ismej.2010.18
